# Impact of Extranodal Extension on Risk Stratification in Papillary Thyroid Carcinoma

**DOI:** 10.1089/thy.2018.0541

**Published:** 2019-07-17

**Authors:** Hye In Kim, Jiyeon Hyeon, So Young Park, Hyeon Seon Ahn, Kyunga Kim, Ji Min Han, Ji Cheol Bae, Jung Hee Shin, Jee Soo Kim, Sun Wook Kim, Jae Hoon Chung, Tae Hyuk Kim, Young Lyun Oh

**Affiliations:** ^1^ Division of Endocrinology and Metabolism, Department of Medicine, Samsung Changwon Hospital, Sungkyunkwan University School of Medicine, Changwon, Republic of Korea.; ^2^ Department of Pathology, Incheon St Mary's Hospital, College of Medicine, The Catholic University of Korea, Seoul, Republic of Korea.; ^3^ Division of Endocrinology and Metabolism, Department of Medicine, Thyroid Center, Samsung Medical Center, Sungkyunkwan University School of Medicine, Seoul, Republic of Korea.; ^4^ Statistics and Data Center, Research Institute for Future Medicine, Samsung Medical Center, Sungkyunkwan University School of Medicine, Seoul, Republic of Korea.; ^5^ Department of Radiology, Samsung Medical Center, Sungkyunkwan University School of Medicine, Seoul, Republic of Korea.; ^6^ Division of Breast and Endocrine Surgery, Department of Surgery, Samsung Medical Center, Sungkyunkwan University School of Medicine, Seoul, Republic of Korea.; ^7^ Department of Pathology, Samsung Medical Center, Sungkyunkwan University School of Medicine, Seoul, Republic of Korea.

**Keywords:** papillary thyroid carcinoma, recurrence, extranodal extension, risk-stratification system

## Abstract

***Background:*** The current American Thyroid Association risk-stratification system for papillary thyroid carcinoma (PTC) incorporates the number and size of positive lymph nodes (LNs) but places less weight on extranodal extension (ENE). This study investigated how to incorporate ENE into the current system to predict recurrence better in PTC N1 patients.

***Methods:*** A total of 369 N1 PTC patients without distant metastasis were enrolled. The combination of number of positive LNs and LNs with ENE that had the highest C-index were identified with multivariable Cox proportional hazards models. ENE number was incorporated into the current system considering the recurrence rate and unadjusted and adjusted hazard ratios (HRs) of the subgroups. Kaplan–Meier curves for recurrence based on current and alternative systems were compared by log-rank test.

***Results:*** The recurrence rate for the subgroup with five or fewer positive LNs and one to three ENEs (7/61; 11.5%) was higher than that of the subgroup with five or fewer positive LNs without ENE (5/129; 3.9%; adjusted HR = 3.42 [confidence interval (CI) 0.99–11.75]; *p* = 0.050). In contrast, adjusted HRs of the subgroup with more than five positive LNs and one to three ENEs (2.33 [CI 0.52–10.35]) or with four or more ENEs (3.86 [CI 1.05–14.17]) were not higher than those of the subgroup with more than five LNs without ENE (4.47 [1.16–17.19]). Incorporating ENE into the current system as an intermediate-risk group yielded a lower log-rank *p*-value (0.05 vs. 0.01) than the current system.

***Conclusions:*** The presence of ENE in low volume LN metastasis confers an intermediate risk of recurrence. Incorporating ENE into the current system allows more accurate decisions regarding further management of PTC N1 patients.

## Introduction

Physicians currently use the risk-stratification system of the American Thyroid Association (ATA) to determine further management options for patients with papillary thyroid carcinoma (PTC), including radioactive iodine (RAI) treatment, degree of thyrotropin (TSH) suppression, and surveillance. The currently used system incorporates two lymph node (LN) criteria to stratify nodal disease: the number and largest size of positive LNs (1).

The presence of extranodal extension (ENE), which is a well-known adverse prognostic factor for various cancer types (2–4), has emerged as an important risk factor for the recurrence of PTC (5–8). Furthermore, Leboulleux *et al*. suggested that the number of ENEs is associated with an increased risk of recurrence of PTC (9). Based on this study, the ATA guidelines state that patients with LNs with three or fewer ENEs are at low risk of recurrence (2%), while those with LNs with more than three ENEs are at high risk of recurrence (about 40%) (1).

However, ENE was not incorporated in the current ATA risk-stratification system for recurrence for the following reasons. First, the presence and number of ENEs are closely linked to the number of positive LNs, which is already included as a variable in the system (1,10). In addition, the lack of definition of an ENE, the high average number of LNs with ENE, and no evaluation of largest LN size in a previous study (9) made it difficult to incorporate ENEs into the current guidelines. Considering the prognostic significance of ENE, an alternative ATA risk-stratification system that includes ENE criteria is needed. However, most studies have focused only on the fact that ENE is a risk factor of recurrence, and have not investigated how to incorporate ENE into the current ATA risk-stratification system taking multi-collinearity into consideration.

Accordingly, this study evaluated the impact of the presence and number of ENEs on recurrence in patients with PTC, with an emphasis on how to incorporate this information into the current ATA risk-stratification system.

## Methods

### Study subjects

Records of 958 patients with PTC who underwent total thyroidectomy and RAI treatment between April 1, 2012, and December 31, 2014, at the Samsung Medical Center were reviewed retrospectively. Exclusion criteria included age <18 years at time of surgery (*n* = 5), N0 disease (*n* = 113), tumor size ≤1.0 cm (*n* = 366), and lack of available data for LNs (*n* = 105). A total of 369 patients were enrolled in the present study (Supplementary Fig. S1). This study was approved by the Institutional Review Board of the Samsung Medical Center (IRB no. 2018-02-034).

### Evaluation of LNs

**FIG. 1.**
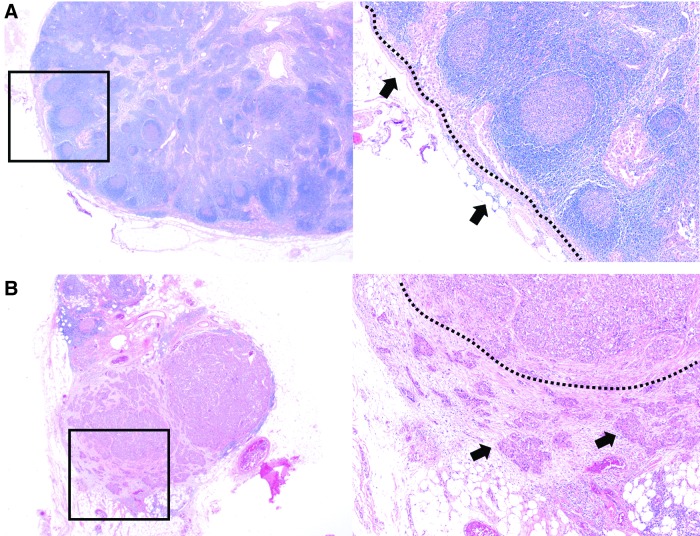
Example of lymph nodes (LNs) negative (**A**) or positive (**B**) for extranodal extension (ENE) in patients with papillary thyroid cancer (PTC; hematoxylin and eosin stain; original magnification × 10 and × 40). (**A**) ENE negative: no tumor cell invading the perinodal soft tissue (arrows) and an intact nodal capsule (dotted line). (**B**) ENE positive: tumor cells invading the perinodal soft tissue (arrows) beyond the nodal capsule (dotted line).

All enrolled patients underwent central compartment node dissection with or without lateral compartment node dissection. According to the surgeon's judgment based on the ATA guidelines, both prophylactic central neck dissection (CND) and selective CND were performed (1,11). In the case of prophylactic CND, usually an ipsilateral paratracheal dissection was performed. A bilateral paratracheal dissection was performed only if there was documented contralateral paratracheal LN metastasis or N1b disease. “Berry picking” resection, in which just grossly positive LNs are excised (12), was not performed in any of the enrolled patients. All harvested LNs were fixed in 10% phosphate-buffered formalin, embedded in paraffin, and split evenly. Pathologic slides stained with hematoxylin and eosin were reviewed by two experienced pathologists. In addition to size and total number of positive LNs, the presence and number of ENEs were also evaluated. ENE was defined as the extension of metastatic cells beyond the nodal capsule into the perinodal soft tissue, which is the definition used in several earlier studies (Fig. 1) (2,3,6). The extent or diameter of individual ENEs was not measured.

### Study design and statistical analysis

The outcome of the current study was structural persistent/recurrent disease, which was defined as cytopathology-proven disease or biochemically incomplete evidence (basal serum thyroglobulin >1.0 ng/mL) with a lesion highly suspicious of being recurrent on two serial imaging studies (thyroid ultrasonography, neck computed tomography [CT], whole-body RAI scan, or positron emission tomography-CT). This definition thus referred to both recurrent and biochemically persistent disease that was newly identified in imaging studies. Recurrence-free survival was defined as the time interval (in months) between the initial surgery date and the most recent follow-up date for patients without structural persistent/recurrent disease (13–17).

To resolve the multi-collinearity problem, possible combinations of positive LN number and ENE number were derived. For this, two fixed categories (≤5 positive LNs and >5 positive LNs) for LN number and three non-fixed categories (no ENE, 1 – *K* ENEs, **≥***K* + 1 ENEs) for ENE number were serially matched. *K* is an arbitrary natural number ranging from 2 to 10, and it is used to specify non-fixed categories of ENEs. A total of nine combinations each composed of six subgroups were derived (Table 2).

Cox proportional hazards models were constructed to assess the association between the various combinations and recurrence, with or without adjusting for conventional prognostic factors such as age, sex, gross extrathyroidal extension (ETE), and therapeutic RAI (defined as a RAI dosage of ≥100 mCi) (1). Then, the C-index of the Cox proportional hazards models was compared to identify the best combination for predicting recurrence. The C-index is a statistical method quantifying the goodness of models with binary outcome, which is equal to the area under the receiver operating characteristic (ROC) curve. The combination associated with the highest C-index in the multivariable Cox proportional hazard models was selected (18–20). Last, by comparing recurrence rate and unadjusted and adjusted hazard ratios (HRs) for the risk of structural persistent/recurrent disease of subgroups, the effect of ENE when incorporated into the current ATA risk-stratification system was estimated. The Kaplan–Meier curve for recurrence derived from the proposed system was compared with that of the current system by the log-rank test.

All categorical variables are presented as numbers and percentages, while continuous variables are presented as means ± standard deviations (*SD*s) if the variable followed a normal distribution, and medians with interquartile range (IQR) for continuous variables that did not follow a normal distribution. All statistical analyses were performed using SAS v9.4 (SAS Institute, Cary, NC). The level of significance was set at *p* < 0.05 for two-sided tests.

## Results

### Baseline characteristics

Baseline characteristics of the 369 patients are presented in Table 1. The median age was 42.0 years (IQR 33.5–53.0 years), and 63% (*n* = 234) of patients were female. The median largest positive LN size was 0.6 cm (IQR 0.3–1.1 cm), and the median number of positive LNs was 5 (IQR 2–10), respectively. ENE was detected in 193 (52.3%) of the 369 patients, and the median number of ENEs was 3.0 (IQR 1.0–6.0).

**Table 1.** Patient Characteristics

*Characteristic*	
Female, *n* (%)	234 (63%)
Age (years), median (IQR)	42.0 (33.5–53.0)
ETE, *n* (%)	
No ETE	67 (18%)
Minimal or T3b	256 (69%)
T4	46 (12%)
Tumor size (cm), median (IQR)	1.6 (1.3–2.3)
Aggressive histology, *n* (%)	14 (4%)
Total positive LNs, median (IQR)	5 (2–10)
Patients with >5 positive LNs, *n* (%)	166 (48%)
Largest LN size (cm), median (IQR)	0.6 (0.3–1.1)
Presence of ENE, *n* (%)	193 (52.3%)
Therapeutic RAI, *n* (%)	238 (64.5%)
Follow-up length (months), median (IQR)	40 (32–48)

IQR, interquartile range; ETE, extrathyroidal extension; LN, lymph node; ENE, extranodal extension; RAI, radioactive iodine.

### Combination of positive LN number and ENE number

As expected, the numbers of positive LNs and ENEs were closely related (Pearson's correlation coefficient = 0.66, *p* < 0.001; data not shown). Among nine multivariable Cox proportional hazards models with various combinations of the number of positive LNs and ENEs, combination 2 (≤5 LNs without ENEs; ≤5 LNs and 1–3 ENEs; ≤5 LNs and ≥4 ENEs; >5 LNs without ENEs; >5 LNs and 1–3 ENEs; >5 LNs and ≥4 ENEs) had the highest C-index (0.719 ± 0.049; Table 2).

**Table 2.** Possible Combinations of Numbers of Positive Lymph Nodes and Numbers of Lymph Nodes with ENE AND the C-Index

*Combination K*^a^	*Subgroup 1*	*Subgroup 2*	*Subgroup 3*	*Subgroup 4*	*Subgroup 5*	*Subgroup 6*	*C-index*^b^* ± SE*
	*Positive LNs ≤5*			*Positive LNs >5*			
	*Number of ENEs*			*Number of ENEs*			
Combination 1	0	1–2	≥3	0	1–2	≥3	0.711 ± 0.049
**Combination 2**	**0**	**1–3**	**≥4**	**0**	**1–3**	**≥4**	**0.719 ± 0.049**
Combination 3	0	1–4	≥5	0	1–4	≥5	0.710 ± 0.049
Combination 4	0	1–5	≥6	0	1–5	≥6	0.709 ± 0.049
Combination 5	0	1–6	≥7	0	1–6	≥7	0.709 ± 0.049
Combination 6	0	1–7	≥8	0	1–7	≥8	0.713 ± 0.049
Combination 7	0	1–8	≥9	0	1–8	≥9	0.716 ± 0.049
Combination 8	0	1–9	≥10	0	1–9	≥10	0.709 ± 0.049
Combination 9	0	1–10	≥11	0	1–10	≥11	0.717 ± 0.049

Combination 2 indicates the combination with the highest C-index (shown in bold).

^a^ Combination K was derived by matching three categories (0, 1 – *K*, ≥*K* + 1, where *K* ranged from 2 to 10) for ENE number and two fixed categories (≤5 positive LNs, >5 positive LNs) for the number of positive LNs.

^b^ C-index of multivariable Cox proportional hazard model with the combination included as the main variable for recurrence.

*SE*, standard error.

### Impacts of ENE on structural persistent/recurrent disease

During the median follow-up period of 40 months, structural persistent/recurrent disease was observed in 35 (9.49%) patients. The structural persistent/recurrent disease occurred in local LN (*n* = 29; 6 cases in central LN and 23 cases in lateral LN), local soft tissue except LN (*n* = 5), and a distant area (*n* = 1, the lung) (21). The recurrence rate for the subgroup with five or fewer LNs and one to three ENEs (7/61; 11.5%) was higher than that for the subgroup with five or fewer LNs without ENE (5/129; 3.9%; unadjusted HR = 3.19 [confidence interval (CI) 1.01–10.06], *p* = 0.047; adjusted HR = 3.42 [CI 0.99–11.75], *p* = 0.050) and similar to that of the subgroup with more than five LNs without ENE (recurrence rate 6/47; 12.8%; unadjusted HR = 3.64 [CI 1.11–11.95], *p* = 0.033; adjusted HR = 4.47 [CI 1.16–17.19], *p* = 0.028). The latter subgroup is classified as an intermediate-risk group in the current risk-stratification system. Adequate statistical analysis was not available for the subgroup with five or fewer LNs and four or more ENEs because of the small size of this subgroup and lack of outcome data (0/2).

Recurrence rates of the three subgroups with more than five LNs (intermediate risk in the current system) were 12.7% (6/47), 8.1% (4/49), and 14.1% (11/78), respectively. These values are higher than those obtained for the subgroup with five or fewer LNs without ENE. However, unadjusted and adjusted HRs of the subgroup with more than five LNs with one to three ENEs (2.18 [CI 0.58–8.14] and 2.33 [CI 0.52–10.35]) or with four or more ENEs (4.46 [CI 1.59–12.54] and 3.86 [CI 1.05–14.17]) were not higher than those of the subgroup with more than five LNs without ENEs (3.64 [CI 1.11–11.95] and 4.47 [CI 1.16–17.19]; Table 3).

**Table 3.** Univariable and Multivariate Cox Proportional Hazard Models to Predict Structural Persistent/Recurrent Disease in PTC N1 Patients

*Variable*	*Univariable*		*Multivariable*	
	*HR [CI]*	p*-Value*	*HR [CI]*	p*-Value*
Age ≥55 years	1.18 [0.53–2.60]	0.673	1.07 [0.45–2.53]	0.877
Female	0.60 [0.31–1.17]	0.139	0.74 [0.36–1.52]	0.414
Tumor size (cm)	1.51 [1.15–1.99]	0.003	1.37 [1.00–1.87]	0.049
ETE				
No ETE	Ref.		Ref.	
Microscopic ETE	2.46 [0.74–8.12]	0.138	2.25 [0.67–7.55]	0.186
Gross ETE	2.52 [0.60–10.57]	0.204	1.88 [0.42–8.33]	0.404
Aggressive histology	0.77 [0.10–5.64]	0.798	0.57 [0.07–4.74]	0.610
Therapeutic RAI	1.34 [0.64–2.80]	0.426	1.66 [0.77–3.59]	0.193
N1b disease	1.62 [0.83–3.15]	0.154	1.00 [0.45–2.24]	0.990
Largest LN size (cm)				
<0.2 cm	Ref.		Ref.	
0.2–3.0 cm	1.55 [0.37–6.51]	0.544	0.62 [0.12–3.17]	0.569
≥3.0 cm	21.99 [3.06–157.76]	0.002	2.45 [0.22–26.87]	0.461
Combinations				
≤5 LNs, no ENE	Ref.		Ref.	
≤5 LNs, 1–3 ENEs	3.19 [1.01–10.06]	0.047	3.42 [0.99–11.75]	0.050
≤5 LNs, ≥4 ENEs	—	0.983	—	0.989
>5 LNs, no ENE	3.64 [1.11–11.95]	0.033	4.47 [1.16–17.19]	0.028
>5 LNs, 1–3 ENEs	2.18 [0.58–8.14]	0.244	2.33 [0.52–10.35]	0.265
>5 LNs, ≥4 ENEs	4.46 [1.59–12.54]	0.004	3.86 [1.05–14.17]	0.041

HR, hazard ratio; CI, confidence interval.

### Incorporating ENE into the ATA risk-stratification system

**FIG. 2.**
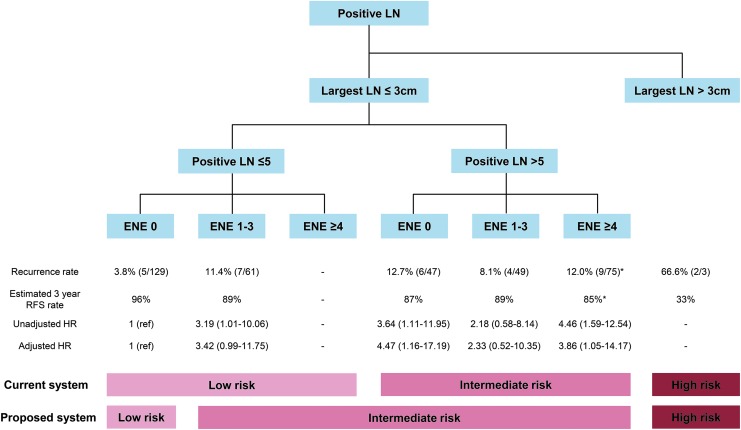
The process of incorporating ENE number into the current American Thyroid Association (ATA) risk-stratification system. *Recurrence rate and estimated three-year RFS rate were calculated after excluding the three patients with the largest LNs (LNs >3 cm) HR, hazard ratio; RFS, recurrence-free survival.

**FIG. 3.**
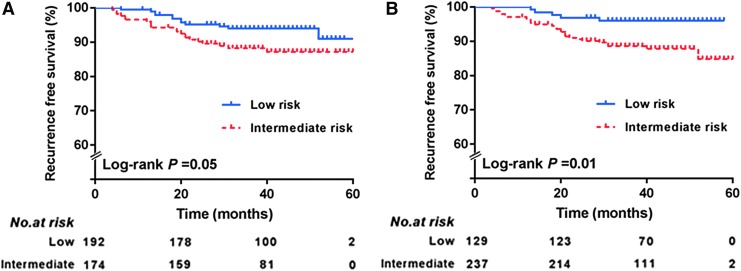
Kaplan–Meier curves for structural persistent/recurrent disease in PTC N1 patients according to (**A**) the current ATA risk stratification system and (**B**) the alternative system.

The process of incorporating ENE into the current risk-stratification system considering the recurrence rate and unadjusted and adjusted HRs of each subgroup is illustrated in Figure 2. Given the similar recurrence rate and unadjusted or adjusted HR, a considerable portion of patients with five or fewer LNs (63/192; 32.8%) but with ENE were up-staged from the low- to intermediate-risk group. Patients who had more than five LNs with ENE remained in the intermediate-risk group because there was no further increase in risk of recurrence according to the presence or number of ENEs. The alternative risk-stratification system showed a significant log-rank *p*-value (*p* = 0.010) for the Kaplan–Meier curve for recurrence, while the current ATA risk-stratification system did not (*p* = 0.050; Fig. 3).

## Discussion

This study defined the optimal combination of number of ENEs and number of positive LNs composed of six subgroups (≤5 LNs without ENE; ≤5 LNs with 1–3 ENEs; ≤5 LNs with ≥4 ENEs; >5 LNs without ENE; >5 LNs with 1–3 ENEs; >5 LNs with ≥4 ENEs) to predict the risk of recurrent/persistent disease better. The subgroup with five or fewer LNs and one to three ENEs had a higher risk of recurrence than the subgroup with five or fewer LNs without ENE, and the risk of recurrence in the subgroup with five or fewer LNs and one to three ENEs was similar to that of the three subgroups with more than five LNs. The presence of ENEs therefore translates to intermediate risk within the current risk-stratification system.

When considering previous studies (5–7,9,22–25), including a recent meta-analysis (8), it seems clear that the presence of ENE is a significant adverse prognostic factor in thyroid cancer. However, the presence and number of ENEs are closely linked with the number of positive LNs, which makes it difficult to incorporate ENEs into the current risk-stratification system. Recent ATA guidelines therefore commented that PTC patients with more than three ENEs have a high risk of recurrence, but this is not reflected in the risk-stratification system (1). In contrast to prior studies, this study combined ENE number and positive LN number using the C-index of multivariable Cox proportional hazards models, which made it possible to evaluate ENE risk within the current risk-stratification system. It was found that stratifying the number of ENEs as 0, 1–3, or ≥4 was most appropriate for predicting structural persistent/recurrent disease in the current risk-stratification system.

Among patients with five or fewer positive LNs (low-risk group in the current system), the presence of ENE had an impact on the risk for structural persistent/recurrent disease of PTC, and HRs were similar to those of subgroups with more than five LNs. Furthermore, despite the presence of five or fewer LNs, the recurrence rate in the subgroup with ENE was >10%, similar to findings for many intermediate groups based on ATA guidelines (minor ETE, 8%; intrathyroidal PTC <4 cm with *BRAF* mutation, 10%; pathologic positive LN number >5, 20%) (1). This result is in accordance with other studies that reported recurrence rates of 14.5% (22), 15% (26), and 22.8% (27) in PTC patients with ENE. In contrast, Leboulleux *et al*. reported a recurrence rate of only 4% in patients with one to three ENEs. However, this result was obtained based on the analysis of only a small number of patients (*n* = 23) with one recurrence. Furthermore, the patients enrolled in that study had an extraordinary high number of ENEs (median 8; IQR 1–57), and ENE was not further defined (9). Considering the prognostic significance of ENE, a detailed pathological examination to determine the presence of ENE should be performed. If ENE is detected, even if individuals have five or fewer positive LNs, patients should be considered to be in the intermediate-risk group. These patients may benefit from further treatment such as RAI treatment or additional TSH suppression.

Interestingly, the impact of ENE on recurrence differed according to the number of positive LNs, and disappeared in patients with more than five positive LNs. Similar to the results of the present study, ENE number in cancers of the oral cavity had a different impact according to the number of positive LNs. In a newly proposed nodal staging system based on recursive partitioning analysis, ENE was retained as a factor only in the case of a single positive LN, and had no impact on survival in patients with carcinomas of the oral cavity with more than two positive LNs (28). Although the exact reason why ENE affects recurrence only in patients with a few positive LNs is unclear, it may be that ENE promotes the spread of nodal disease at an early stage of N1 disease. Also, given that patients with intermediate risk usually receive RAI treatment, the assumption that PTC N1 patients with ENE may show a better response to RAI treatment could be another explanation. This notion is supported by a recent study on pancreatic cancer that demonstrated a better prognosis of patients with ENE when treated with adjuvant chemoradiation therapy but not chemotherapy alone (29). In addition, all three patients with the largest LNs (≥3 cm; high-risk group in the current system) in this study were in the subgroup with more than five LNs and four or more ENEs. This suggests that the extremely high recurrence rate in patients with ENE in the previous study (recurrence rate of 32% in patients with >3 ENEs) (9) may have been influenced by LN size, which was not reported in that study.

Altogether, the presence of ENE appears to equate to an intermediate risk of recurrence within the current ATA system. After incorporating ENE into the current risk-stratification system to create an alternative system, this system showed a better predictive ability than the current ATA risk-stratification system for predicting structural persistent/recurrent disease. The present results are useful when determining the initial treatment after surgery. However, the current study has several limitations that need to be taken into consideration. First, the results of this study might not apply to the long-term prognosis because of the relatively short follow-up period. However, early recurrence can provide enough information to determine the extent of initial treatment. Second, there were too few patients with five or fewer LNs and four or more ENEs to analyze whether ENE had a further impact on recurrence. Third, the possibility that structural persistent/recurrent disease could result from incomplete LN dissection cannot be excluded, although experienced surgeons carried out the operations according to established guidelines (30). The retrospective study design and the single-center nature of the study are additional limitations. Further research is therefore needed to confirm the findings. In addition, although the degree of individual ENE was not evaluated, evaluating a possible association between the degree of ENE and recurrence risk also seems to be a relevant topic for future studies.

In conclusion, the presence of ENE translates into an intermediate risk of recurrence in the current risk-stratification system. By incorporating ENE into the current risk-stratification system, physicians can make more accurate decisions about how to manage PTC N1 patients.

## Supplementary Material

Supplemental data
