# Supplementary figures and images for: Impact of Extranodal Extension on Risk Stratification in Papillary Thyroid Carcinoma
Source: Thyroid. 2019 Jul 17;29(7):963–70. doi: 10.1089/thy.2018.0541 (PMC6648218; doi:10.1089/thy.2018.0541)

## Supplementary Data

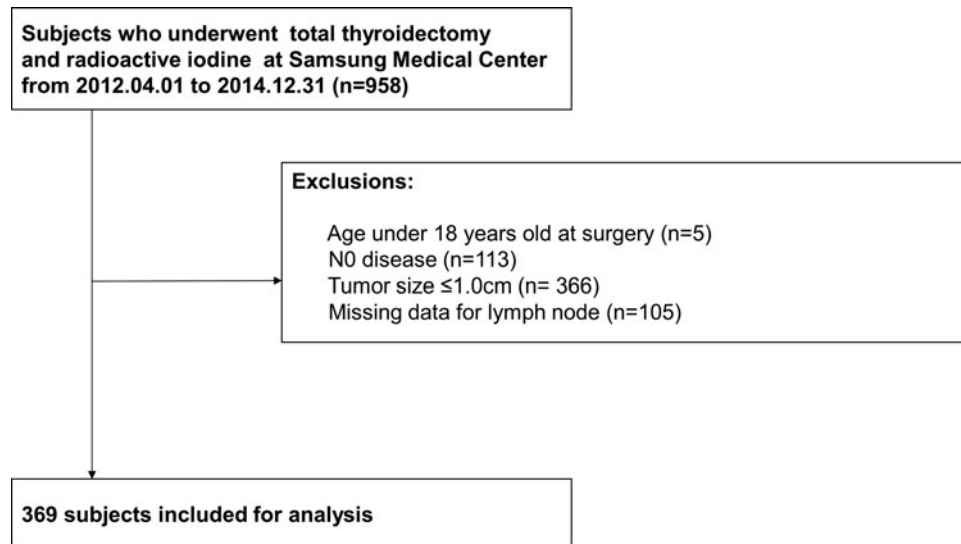

**SUPPLEMENTARY FIG. S1.** Flow chart of patient enrollment.

Supplement: Supplemental data [file Supp_Fig1.pdf]
